# GRACKLE: an interpretable matrix factorization approach for biomedical representation learning

**DOI:** 10.1093/bioinformatics/btaf213

**Published:** 2025-07-15

**Authors:** Lucas A Gillenwater, Lawrence E Hunter, James C Costello

**Affiliations:** Department of Pharmacology, University of Colorado Anschutz Medical Campus, Aurora, CO, 80045, United States; Computational Bioscience Program, University of Colorado Anschutz Medical Campus, Aurora, CO, 80045, United States; Linda Crnic Institute for Down Syndrome, University of Colorado Anschutz Medical Campus, Aurora, CO, 80045, United States; Department of Pediatrics, University of Chicago, Chicago, IL, 60637, United States; Department of Pharmacology, University of Colorado Anschutz Medical Campus, Aurora, CO, 80045, United States; Computational Bioscience Program, University of Colorado Anschutz Medical Campus, Aurora, CO, 80045, United States; Linda Crnic Institute for Down Syndrome, University of Colorado Anschutz Medical Campus, Aurora, CO, 80045, United States; Department of Biomedical Informatics, University of Colorado Anschutz Medical Campus, Aurora, CO, 80045, United States

## Abstract

**Motivation:**

Disruption in normal gene expression can contribute to the development of diseases and chronic conditions. However, identifying disease-specific gene signatures can be challenging due to the presence of multiple co-occurring conditions and limited sample sizes. Unsupervised representation learning methods, such as matrix decomposition and deep learning, simplify high-dimensional data into understandable patterns, but often do not provide clear biological explanations. Incorporating prior biological knowledge directly can enhance understanding and address small sample sizes. Nevertheless, current models do not jointly consider prior knowledge of molecular interactions and sample labels.

**Results:**

We present GRACKLE, a novel nonnegative matrix factorization approach that applies Graph Regularization Across Contextual KnowLedgE. GRACKLE integrates sample similarity and gene similarity matrices based on sample metadata and molecular relationships, respectively. Simulation studies show GRACKLE outperformed other NMF algorithms, especially with increased background noise. GRACKLE effectively stratified breast tumor samples and identified condition-enriched subgroups in individuals with Down syndrome. The model's latent representations aligned with known biological patterns, such as autoimmune conditions and sleep apnea in Down syndrome. GRACKLE's flexibility allows application to various data modalities, offering a robust solution for identifying context-specific molecular mechanisms in biomedical research.

**Availability and implementation:**

GRACKLE is available at: https://github.com/lagillenwater/GRACKLE.

## 1 Introduction

Dysregulation of established gene regulatory networks (GRNs) can drive disease and chronic conditions (Lee and Young 2013). Accordingly, there are many efforts to identify disease-specific gene signatures for mechanistic understanding and diagnostic purposes (Evangelista *et al.* 2022). However, these disease-specific signatures can be challenging to derive and apply, in part because most people experience several chronic conditions simultaneously. Approaches to identify disease-specific gene signatures in the presence of other co-occurring conditions include increasing the sample size, which is not always possible, or adjusting the data for disease confounders, which may remove the biological signal (Li *et al.* 2014). Thus, there is a need for methods that address the complexity of molecular interactions while considering the interdependencies of co-occurring conditions.

Unsupervised representation learning methods aim to reduce high-dimensional data to meaningful and interpretable patterns and structures. This is done by projecting the input data to lower dimensions that capture the dominant components of variation. These lower dimensional representations, or latent variables (LVs), can then be tested for enrichment of biological pathways, processes, or other annotations that might help explain the variation in a given LV. Common approaches include matrix decomposition [e.g. principal components analysis (PCA), nonnegative matrix factorization (NMF)] and deep learning (e.g. autoencoders) (Ravindran and Gunavathi 2023). A benefit of matrix decomposition over autoencoders is the direct interpretation of the molecular patterns in the context of sample annotation (i.e. cell type, disease state, etc.) due to the alignment between LVs of the decomposed sample and the molecular feature matrix (i.e. loadings) (Johnson *et al.* 2023).

Graph regularized Nonnegative Matrix Factorization (GNMF) extends the matrix factorization framework to consider the local invariance assumption in calculating LVs, i.e. the expectation that similar samples or molecular features will have similar latent representations (Cai *et al.* 2011). Geometric information of the input data is represented by an affinity graph [or graphs in the case of graph dual regularized NMF (DNMF)] of learned data similarities (e.g. *k*-nearest neighbors, cosine similarity) and incorporated into matrix decomposition through regularization (Shang *et al.* 2012). Previous studies demonstrate that variants of GNMF and DNMF outperform NMF in gene clustering tasks (Cai *et al.* 2011, Zhu *et al.* 2017, Yu *et al.* 2018, Jiao *et al.* 2020). Nevertheless, unsupervised representation learning approaches, including GNMF and DNMF, only consider statistical relationships within the training data itself and interpretation is performed *post hoc* with no guarantee that outputs will result in meaningful biological understanding.

To address the challenges of interpretation in the unsupervised setting, prior knowledge in the form of structured biological data can be included directly into the models themselves to better align the model output with established biological knowledge. This approach increases interpretability and can also address the challenges of small sample size (Crawford and Greene 2020). The Pathway Level Information ExtractoR (PLIER) model is an example of directly including pathway information in the decomposition of gene expression matrices. The model outputs, specifically the LVs, align with a constrained number of pathways. This approach of regularizing the matrix decomposition with respect to prior pathway information was shown to improve interstudy reproducibility and outperform methods that estimate cell proportions from bulk transcription profiles (Mao *et al.* 2019). There have been multiple extensions of PLIER to improve the power of rare disease studies, to contextualize genetic associations, and to identify gene programs mediating the effects of karyotype on disease (Taroni *et al.* 2019, Pividori *et al.* 2023, Nandi *et al.* 2025).

Network models of known gene–gene interactions provide a scaffold for ensuring that connected genes are similarly represented in dimension reduction algorithms. For example, in netNMF-sc, regularization by known gene–gene interactions improved performance on the tasks of single cell clustering and gene imputation (Elyanow *et al.* 2020). Moreover, identifying phenotype-specific representations is crucial for mechanistic inference in clinically relevant settings. For example, PhENotype-assoCiated subpopulatIons from single-celL data (PENCIL) performs feature selection with prediction functions to identify subpopulations specifically associated with clinical phenotypes (Ren *et al.* 2023).

Approaches to align model outputs with prior knowledge have considered either known molecular interactions (e.g. pathway, gene–gene interactions) or sample labels (e.g. phenotypes or other sample annotation), but do not consider both simultaneously. Instead, models incorporating one form of prior knowledge are evaluated using the other. For example, PLIER incorporates pathway information to decompose gene expression data, then the model output was evaluated based on the accuracy of predicting relative cell type proportions (Mao *et al.* 2019). Interestingly, the GNMF framework presents an opportunity to identify latent spaces that incorporate prior information on samples and molecular features simultaneously. The output of such a method could identify subgroup-specific pathways and processes. For example, while leveraging clinical annotation and an established molecular interaction network, a gene expression matrix of patient samples could be decomposed into a lower dimensional representation where the LVs map to a clinically meaningful patient stratification (e.g. molecular subtypes, treatment response) and the genes within each LV are enriched for molecular interactions specific to the associated patient stratification. Such an approach would have an array of applications, including identifying context-specific molecular mechanisms for precise diagnostic and treatment approaches, even in small and heterogeneous cohorts.

Here, we present a novel NMF approach that applies Graph Regularization Across Contextual KnowLedgE (GRACKLE) to learn latent representations. GRACKLE constrains the unsupervised NMF model using both sample similarity and gene similarity matrices. The model is flexible to incorporate multiple forms of sample metadata, such as phenotype or subtype labels, as well as molecular relationships, such as gene–gene interactions or pathway annotation. We demonstrate the efficacy of GRACKLE first through simulation studies, second in defining molecular subtypes in breast cancer, and finally in the deconvolution of individuals with Down syndrome across multiple co-occurring conditions.

## 2 Materials and methods

### 2.1 Simulation, cancer, and Down syndrome datasets

For the simulation study, we generated a GRN and leveraged a gene expression simulation framework to generate gene expression profiles. For the GRN, we used a previously generated transcription factor (TF)-GRN that was inferred from breast tissue gene expression profiles from the GTEx database using Passing Attributes between Networks for Data Assimilation (PANDA) (Glass *et al.* 2013, Lonsdale *et al.* 2013). The adjacency matrix was downloaded from the database of GRNs across human conditions (Ben Guebila *et al.* 2022). An edge between TF i and gene j indicates that there is evidence that i transcriptionally regulates j. The edge weight represents the probability of the regulatory relationship based on the integration of gene expression data, protein–protein interaction data, and TF binding data. To generate a directed network, which is needed for gene expression profile simulation, we transformed the directed network to only include high probability edges (weight > 1) such that,


(1) IGRN(wij)={1 if wij>1,0 otherwise,

where $I_{\mathrm{GRN}}$ is the indicator function and $w_{ij}$ represents the edge weight between TF i and gene j.

To simulate gene expression profiles, we used the Stochastic Gene Network Simulator (sgnesR package version 0.90.1), which applies the Stochastic Gene Network Simulator (SGNSim) in the R environment (Ribeiro and Lloyd-Price 2007, Tripathi *et al.* 2017). The SGNSim framework takes a GRN as input and simulates static or time-series gene expression profiles based on multiple time-delayed events including translation rate, RNA degradation rate, protein degradation rate, TF unbinding rate, and transcription rate, with the stochasticity observed in biological regulation. We used the default rates for the time-delayed events: 0.002, 0.005, 0.005, 0.005, 0.01, 0.02, respectively. To simulate static gene expression that is representative of experimental gene expression profiles (i.e. at different points of the cell cycle), we randomly sampled between 400 and 600 simulator iterations for each sample and took the average of three simulations. Simulated expression profiles were min–max scaled before they were used as input to each of the algorithms.

In addition to the simulations, we identified modules in the GRN that we systematically upregulated in a set of sample profiles. These adjustments are intended to simulate subgroup-specific gene regulation activation. We identified the community structure of the network with the Louvain clustering algorithm using the igraph R package (version 2.1.3) (Blondel *et al.* 2008, Csárdi *et al.* 2025). We randomly selected five modules from the identified communities and simulated the upregulation of genes in a module in a subset of corresponding sample profiles. We assigned phenotypes to simulated samples based on the upregulated module. Finally, to assess GRACKLE’s performance over noisy gene expression profiles, we added background noise by randomly upregulating (Gaussian noise) defined percentages of genes that were not in corresponding modules.

We assessed the performance of GRACKLE on stratifying samples from The Cancer Genome Atlas Breast Invasive Carcinoma (TCGA-BRCA) into molecular subtypes across three different sample similarity matrices (Koboldt *et al.* 2012). TCGA-BRCA gene expression profiles and clinical annotations were downloaded using the TCGAbiolinks R package (version 2.34.0) (Colaprico *et al.* 2016). Samples lacking hormone-receptor subtype assignments were removed because these subtypes were used to evaluate model performance. Unstranded transcripts per million profiles were filtered for the top 5000 genes with the greatest variance over the samples, then min–max scaled. The same breast tissue GRN used in the simulation studies was also used in this study. The gene expression data and GRN were filtered for overlapping genes, resulting in gene expression profiles for 4738 genes over 1099 tumor samples.

In addition, we evaluated the performance of GRACKLE with regularization from a different GRN and on a different breast cancer dataset. For the different GRN, we tested the effects of the STRING protein–protein interaction network on the TCGA PAM50 regularization (Szklarczyk *et al.* 2023). We filtered the network based on the top 5000 most variable genes in the TCGA-BRCA profiles. This resulted in a network of 4557 genes. We also validated GRACKLE on gene expression profiles from 1980 tumors in the Molecular Taxonomy of Breast Cancer International Consortium (METABRIC) consortium (Koboldt *et al.* 2012). We downloaded the gene expression profiles and corresponding metadata from cBioPortal (Cerami *et al.* 2012). As with the TCGA analysis, we filtered the genes to the top 5000 most variable and then further filtered by overlap with the PANDA breast tissue GRN, which resulted in 4734 genes.

For the Down syndrome (T21) study, whole blood gene expression profiles were downloaded from the Human Trisome Project (HTP) (Galbraith *et al.* 2023). The gene expression data for 244 individuals with T21 was processed and filtered as previously reported (Gillenwater *et al.* 2024). Profiles were further filtered to only include the 5000 genes with the greatest variance over the samples, then min–max scaled. The sample metadata consisted of clinical histories of co-occurring conditions recorded for study participants through a combination of participant/caregiver surveys and annotation of medical records. We selected the six most frequent conditions across the cohort, which included history of anxiety, depression, autoimmune skin conditions, hypothyroidism, sleep apnea, and obesity. We downloaded the whole blood GRN consisting of TF-gene regulatory relationships from the database of GRNs across human conditions (Ben Guebila *et al.* 2022). As with the breast network in the simulation studies, the whole blood GRN was filtered to only include high probability edges using Equation (1). Finally, both the gene expression profiles and GRN were filtered to include overlapping genes, resulting in a network and gene expression matrix of 4795 genes.

### 2.2 Nonnegative matrix factorization (NMF)

NMF factorizes a matrix of nonnegative values, $Y\;\in R^{m\times n}$, for m samples and n features, into two matrices $W\;\in R^{m\times k}$ and $H\;\in R^{k\times n}$, where k≪min⁡{m,n}. such that,


(2) Y∼WH

The nonnegative constraints decompose the matrix into parts that are additive combinations. Each column in Y is approximated by the linear combinations of columns in W weighted by the components of H (Lee and Seung 2000) between two matrices (the square of the Frobenius norm of two matrices difference). Since k is much smaller than the dimensions of Y, a good approximation is achieved if the factorized matrices captures structure that is latent in the data. A common cost function is the square of the Euclidean distance defined as,


(3) $$\min_{W,H}||Y-WH|{|}_{F}^{2},$$

Equation (3) is minimized through iterative multiplicative updates of the W and H matrices. Multiplicative updates are applied using,


(4) $$w_{ik}\leftarrow w_{ik}\times \frac{YH^{T}}{WHH^{T}},\;h_{jk}\leftarrow h_{jk}\times \frac{W^{T}Y}{HWW^{T}},$$

i and j refer to sample and gene, respectively. The computational complexity of each multiplicative update for NMF is O(MNk), where M is the number of observations, N the number of features, and k the number of latent variables. Thus, for the algorithm to converge the total complexity is O(tMNk), where t is the number of iterations.

### 2.3 Graph regularized NMF

Graph regularization in matrix decomposition, such as GNMF and DNMF, learn affinity matrices from the input data to ensure the decomposed matrices consider the local invariance of pairwise relationships (Cai *et al.* 2011, Shang *et al.* 2012). For example, in GNMF a nearest neighbor graph S, which is used to calculate the graph Laplacian as L=D-S, with D representing the diagonal of S, and incorporated into the loss function as:


(5) $${\;\min}_{W,H}||Y-WH|{|}_{F}^{2}+\lambda TR(H^{T}LH)$$

where TR(⋅) is the trace of a matrix, and λ is a tuning parameter for graph regularization. However, there is potential for spurious correlations in the learned affinities (Hsieh *et al.* 2023). Moreover, the learned matrices do not include additional information from matched molecular data, phenotype labels, or curated orthogonal studies. To include prior knowledge of biological interactions, netNMF-sc replaces S with a prior gene–gene interaction network (Elyanow *et al.* 2020). This approach results in more accurate cell clustering and estimations of gene–gene covariance. However, netNMF-sc does not regularize the sample matrix since the method evaluation was performed using the prior sample labels.

### 2.4 GRACKLE

**Figure 1.**
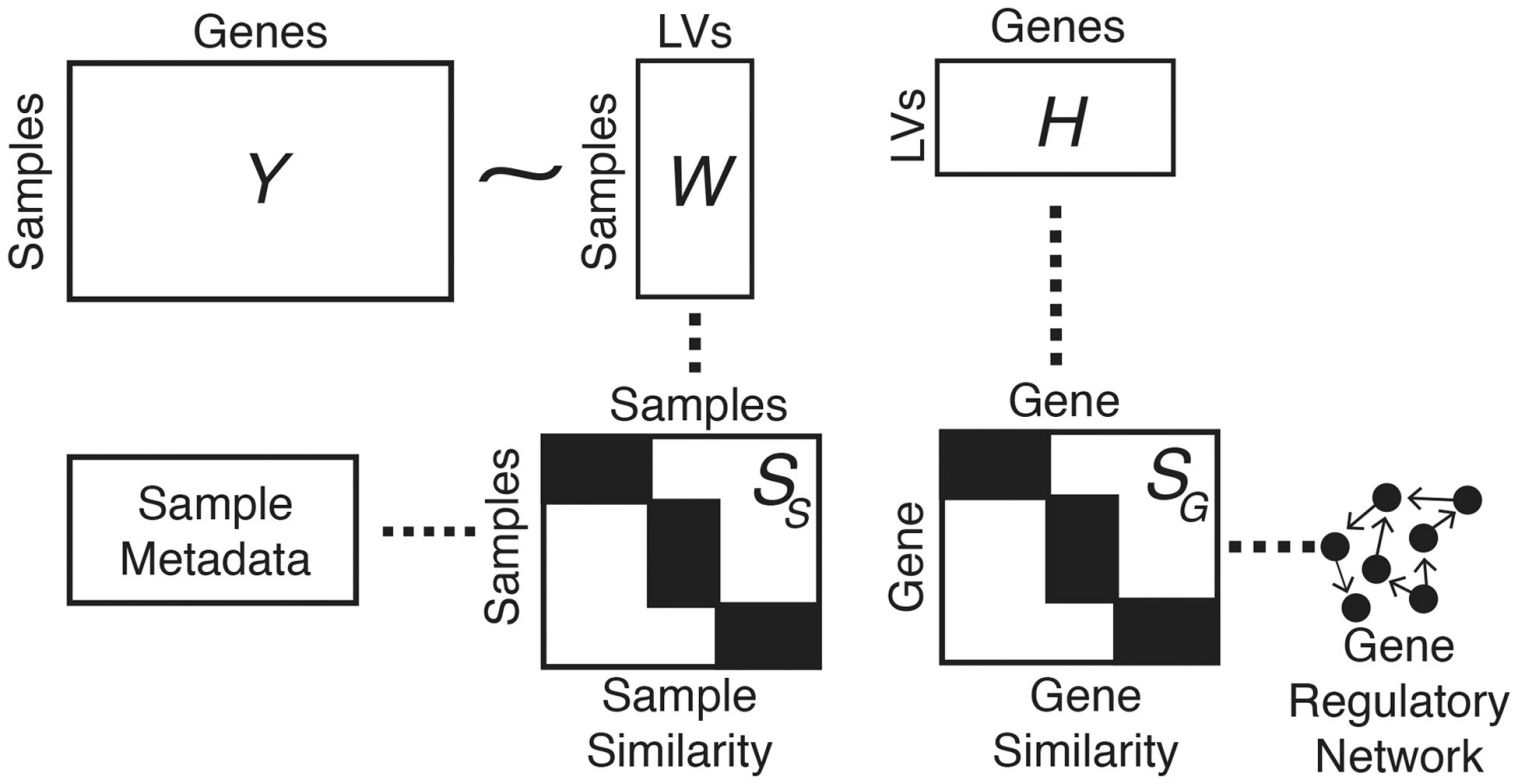
Overview of GRACKLE. The inputs to GRACKLE include a normalized transcript matrix from bulk RNAseq profiling, a matrix of sample metadata which may include sample labels or additional matched omic profiles, and a gene network.

In GRACKLE, we consider external information on the samples like phenotype labels or matched omic profiles, as well as known gene regulatory events (Fig. 1). Before matrix decomposition, the sample metadata is transformed into the sample similarity matrix $S_{S}$ by taking the dot product. Similarly, the gene interactions constitute the gene similarity matrix $S_{G}$. These similarity matrices are used to calculate the graph Laplacians $L_{S}$ and $L_{G}$, which are incorporated into the loss function as,


(6) $$\min_{W,H}||Y-WH|{|}_{F}^{2}+\lambda_{1}TR(W^{T}L_{s}W)+\lambda_{2}TR(H^{T}L_{G}H)$$

where $\lambda_{1}$ and $\lambda_{2}$ are tuning parameters for graph regularization. Accordingly, the multiplicative update functions from Equation (4) are changed to,


(7) $$w_{ik}\leftarrow w_{ik}\times \frac{YH^{T}+\lambda_{1}S_{S}W}{WHH^{T}+\lambda_{1}D_{S}W},\;h_{jk}\leftarrow h_{jk}\times \frac{W^{T}Y+\lambda_{2}S_{G}H}{HWW^{T}+\lambda_{2}D_{G}H}$$

The model is iteratively trained until a stopping criterion of relative change in H is less than 1 × 10^−4^ between iterations or a maximum of 100 iterations.

The computational complexity of GRACKLE depends on the sparsity of similarity matrices used in regularization. If the matrices are sparse, the complexity remains *O*(*tMNk*), as outline by Cai *et al.* (2011) and Shang *et al.* (2012). However, if the similarities are not sparse, then the complexity is constrained by the feature space to *O*(*tN^2^k*).

## 3 Results

### 3.1 Regularization by prior sample similarity improves performance in simulation studies

**Figure 2.**
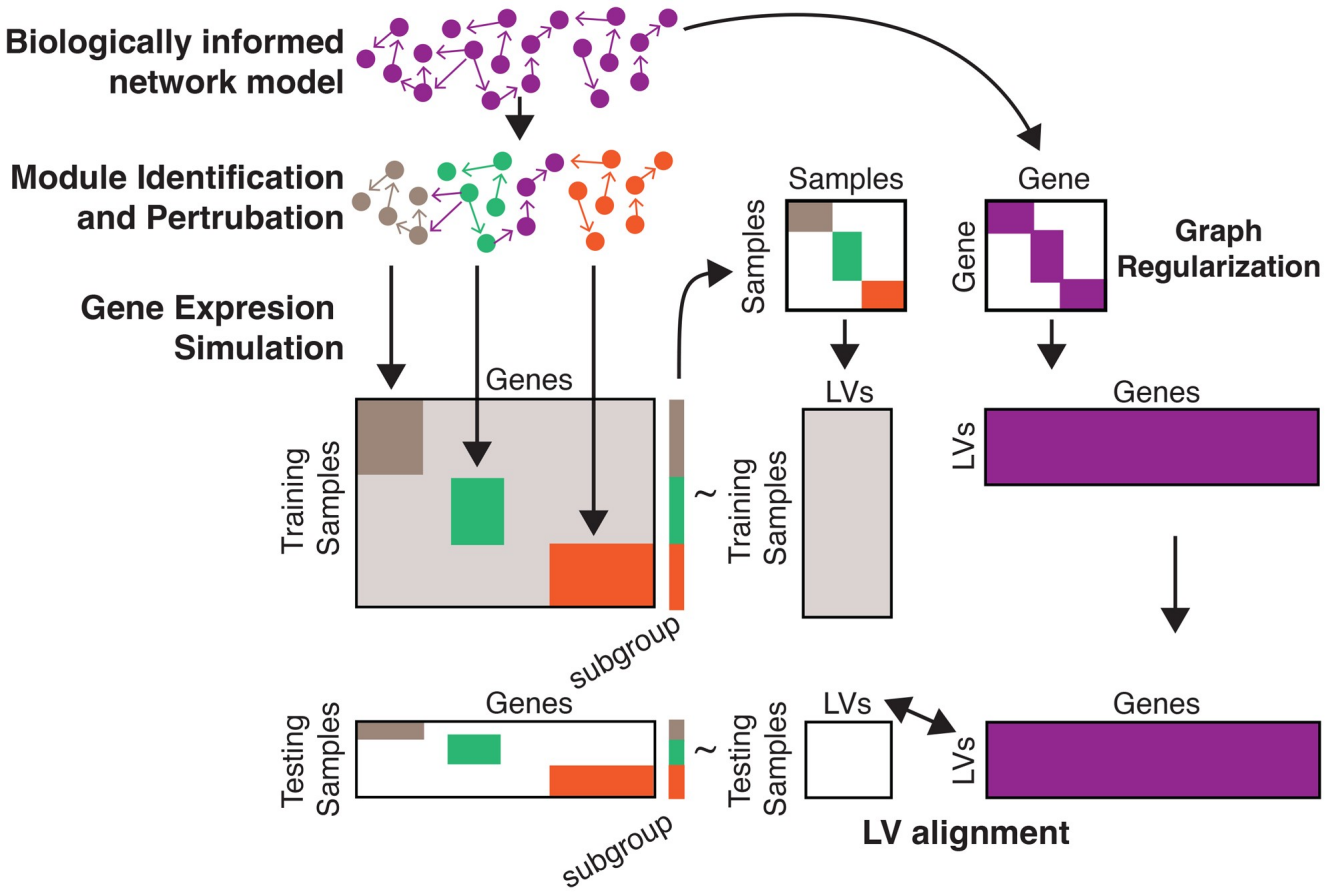
Simulation study design. For simulation studies, a random gene network was generated to match the graph properties of the breast tissue network generated using Passing Attributes between Networks for Data Assimilation (PANDA) (Glass *et al.* 2013, Lonsdale *et al.* 2013). Regulatory modules were identified with the Louvain clustering algorithm using the igraph R package (version 2.1.3) (Blondel *et al.* 2008, Csárdi *et al.* 2025). Simulated gene expression profiles were generated for using the Stochastic Gene Network Simulator (sgnesR package version 0.90.1). Gene modules were specifically perturbed to model pathway upregulation and samples were assigned labels to correspond with perturbations. Simulated profiles were iteratively split into 70/30 training/testing splits for 100 iterations. Training data, sample, and gene similarities were input into GRACKLE. Testing gene expression data were projected into the sample matrix (W) using nonnegative least squares based on the trained gene matrix (H). We determined the accuracy of alignment to the subgroups based on whether the corresponding latent variable (LV) in the decomposed sample and gene matrices had the highest loadings for samples and genes of an assigned subgroup.

To establish a performance benchmark, we compared GRACKLE to other NMF models using simulated gene expression data generated for 400 genes across 100 samples. Illustrated in Fig. 2, the simulated profiles were based on TF-gene regulatory relationships inferred from breast tissue from GTEx using the PANDA algorithm (Glass *et al.* 2013, Lonsdale *et al.* 2013). Gene expression profiles were then simulated using the inferred GRN as input to the SGNSim (Ribeiro and Lloyd-Price 2007, Tripathi *et al.* 2017). In addition, since the aim of GRACKLE is to identify subgroup-specific gene regulatory patterns, we isolated five network modules and systematically upregulated genes in these five modules in a set of samples to simulate subgroup-specific gene regulatory activation.

We assessed the ability of GRACKLE and the other algorithms to decompose the gene expression data into latent variables that matched the simulated subgroups (i.e. gene module differential gene regulation) (Fig. 2). We randomly stratified the simulated profiles into a 70/30 training/testing split, then calculated the decomposed *W* and *H* matrices from the training data. We next projected the testing gene expression data into *W* using nonnegative least squares based on the trained *H* matrix. We calculated the accuracy of alignment to the subgroups based on whether the corresponding latent variable in the decomposed sample and gene matrices have the highest loadings for samples and genes of an assigned subgroup. For example, we consider a result to be accurate if the samples in subgroup A and the corresponding upregulated genes in module A have the highest loadings in LV1 of the decomposed sample and gene matrices *W* and *H*. We reported the average accuracy over all subgroups.

In the simulation studies, we evaluated performance over $\lambda_{1}$ (penalization for sample similarity, $S_{S}$) and $\lambda_{2}$ (penalization for gene similarity, $S_{G}$) values [0, 1] at an interval of 0.1. We tested GRACKLE over varying levels of background gene expression noise, decreased network modularity, and increased network transitivity (i.e. the percent of graph nodes involved in triangles). We benchmarked GRACKLE against three comparable algorithms: NMF, GNMF, and *a prior* informed graph regularized NMF inspired by netNMF-sc, which we call pr-GNMF. The NMF model served as a baseline and corresponds to $\lambda_{1}$ = 0 and $\lambda_{2}$ = 0. For GNMF, the affinity matrix for the gene regularization was calculated with *k*-nearest neighbors and the λ parameter, which affects the degree of graph regularization, was optimized using the parameters defined by Cai *et al.* (number of nearest neighbors is 5, λ = [1, 10, 10^2^, 10^3^, 10^4^] (Cai *et al.* 2011)). For pr-GNMF, the same GRN used for GRACKLE was used for regularization of the gene similarity matrix. For both GNMF and pr-GNMF, graph regularization was performed for the maximum value of $\lambda_{2}$ tested. To avoid overfitting, we calculated the average performance over 100 iterations.

**Figure 3.**
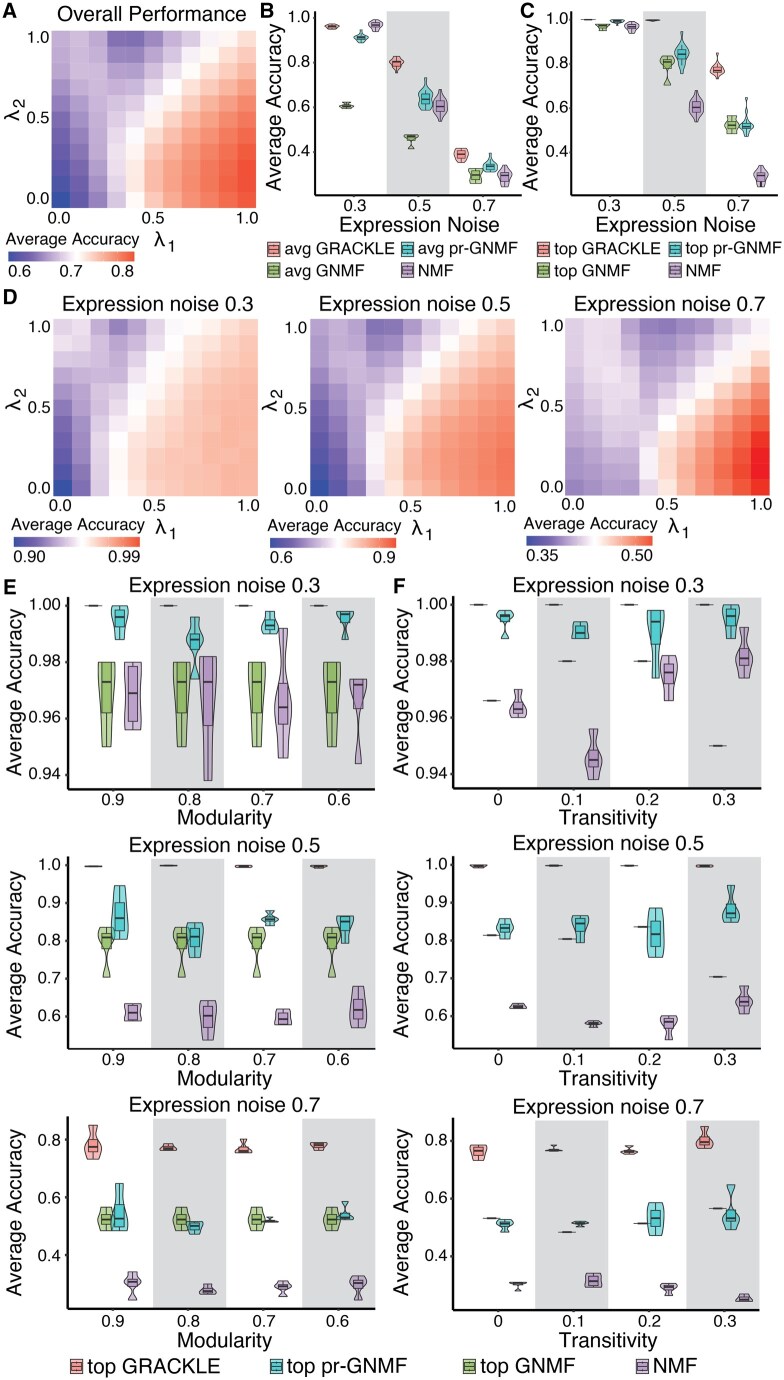
Regularization by prior sample similarity improves performance in simulation studies. (A) Grid search of the overall average accuracy of alignment between the top loadings in sample and gene latent variables over *λ*_1_ and *λ*_2_ parameters over 100 iterations. Reported accuracies are the average over all tested parameters, including background gene expression noise, network modularity, and network transitivity. (B) The overall average accuracy of GRACKLE in comparison to other NMF frameworks at differing levels of background gene expression noise over 100 iterations. (C) Accuracy of the top performing parameter combination for GRACKLE and benchmark algorithms. (D) Grid search of the average accuracy of alignment between the top loadings in sample and gene latent variables over *λ*_1_ and *λ*_2_ parameters over 100 iterations. Reported accuracies are the average at each level of background gene expression. (E–F) The accuracy of top performing GRACKLE in comparison to other NMF frameworks at differing levels of background gene expression noise and network modularity (E) and transitivity (F) over 100 iterations.

Overall, we found that GRACKLE performed best at higher levels of regularization from $S_{S}$ with less background gene expression noise (Fig. 3A). Increased regularization by either $S_{S}$ or $S_{G}$ resulted in improved performance when there was less regularization from the other similarity matrix. However, the performance was significantly improved through increased regularization with respect to $S_{S}$ as compared to $S_{G}$. This trend was observed across increasing background gene expression noise (Fig. 3D). The GRACKLE performance across the range of $\lambda_{1}$ and $\lambda_{2}$ values performed similarly to NMF, GNMF, and pr-GNMF at low levels of background gene expression noise, but outperformed the benchmarks as background noise increased (Fig. 3B). However, when using the optimal $\lambda_{1}$ and $\lambda_{2}\;$parameter set GRACKLE showed much better performance than any other method (Fig. 3C). Decreasing the modularity of the simulated GRN used in the regularization led to a slight decrease in GRACKLE’s optimal parameters and averaged performance, though the effect was not significant (Fig. 3E). Furthermore, increasing the transitivity of the GRN for the simulations did not have a consistent effect across GRACKLE or the other benchmarked algorithms (Fig. 3F).

Taken together, the simulation studies revealed that GRACKLE performs best with increased regularization from $S_{S}$ and that background gene expression noise had a greater effect on performance than GRN modularity or transitivity. Moreover, when the optimal regularization values were used to train GRACKLE, the model strongly outperformed other NMF methods and was robust to noise in the data. GRACKLE performance averaged over the full parameter set also showed superior performance, particularly in the context of noisy data.

### 3.2 GRACKLE identifies breast tumor subtypes when including external sample labels

We assessed the performance of GRACKLE on stratifying samples from The Cancer Genome Atlas Breast Invasive Carcinoma (TCGA-BRCA) into molecular subtypes across three different sample similarity matrices (Koboldt *et al.* 2012). The subtypes used were the gene expression-based “intrinsic” groupings, which includes the five assignments: luminal A, luminal B, HER2-enriched, basal-like, and normal (hereafter referred to as the PAM50 subtypes) (Sørlie *et al.* 2001). These subtypes are well benchmarked and are clinically important for making treatment decisions. To calculate $S_{S}$, we considered three sample metadata labels, including the PAM50 subtypes as a positive control, methylation-based subtypes from an unsupervised clustering analysis, and subtypes generated from unsupervised clustering of matched profiles of lncRNA, methylation, copy number variation, and protein abundance.

As in the simulation studies, we randomly stratified the TCGA-BRCA profiles into a 70/30 training/testing split before projecting the testing gene expression split into the *W* matrix using nonnegative least squares based on the trained *H* matrix. Algorithm performances were evaluated for alignment to the PAM50 subtypes based on the adjusted rand index (ARI) between the latent variable with the top loading per sample and PAM50 subtype assignments (Rand 1971). As a negative control, we randomly permuted the sample metadata labels and recalculated the sample similarity. Finally, we explored algorithm performance over expanded $\lambda_{1}$ and $\lambda_{2}$ parameters ([0, 100] at an interval of 10 and [0, 20] at an interval of 2, respectively). To avoid overfitting, we calculated the average performance over 50 iterations.

**Figure 4.**
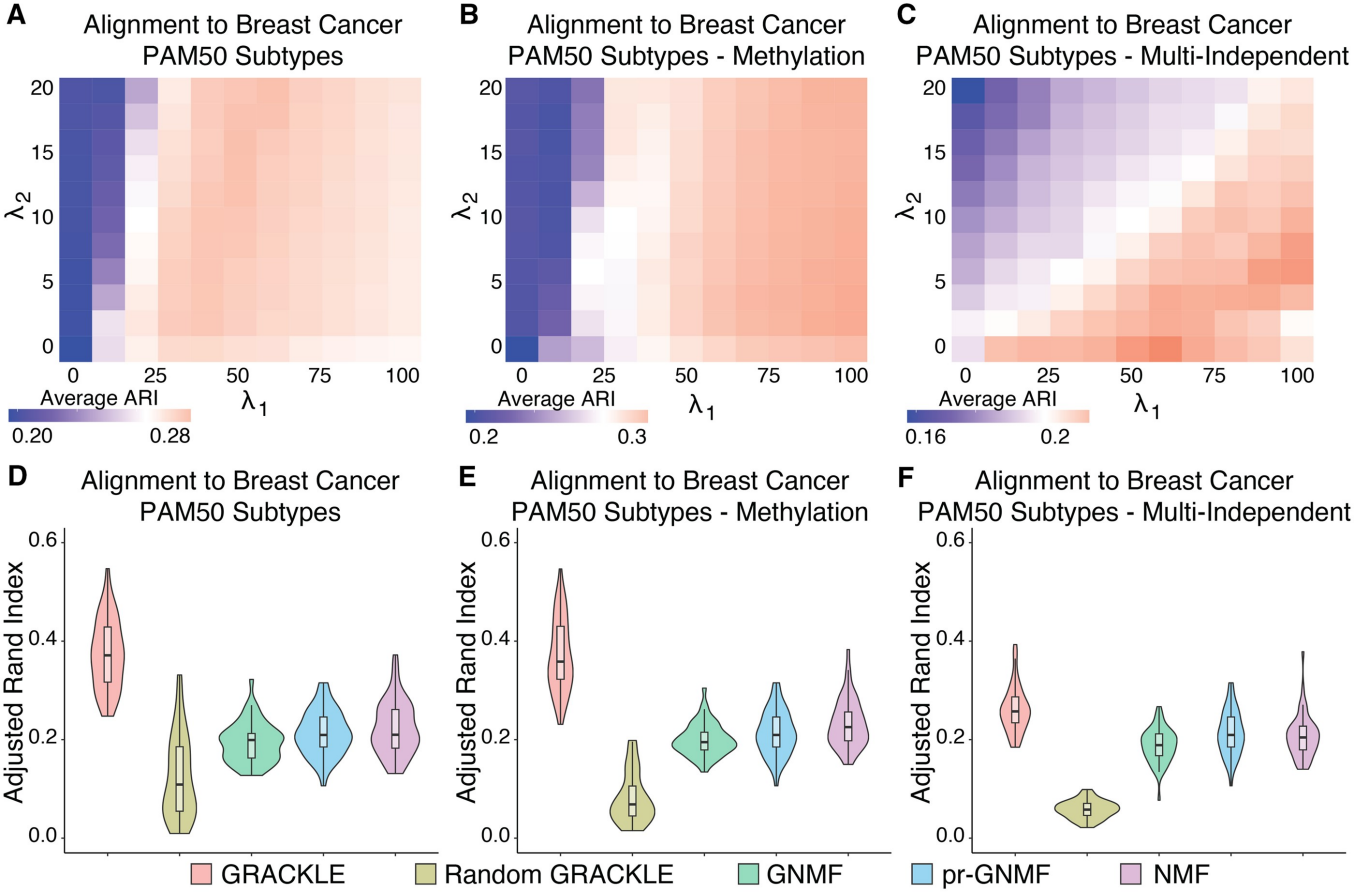
GRACKLE recaptures breast tumor subtypes when including external sample labels. (A–C) Grid search of the average adjusted rand index (ARI) over 50 iterations for alignment between the top loadings in sample latent variables and PAM50 subtypes over *λ*_1_ and *λ*_2_ parameters using regularizing similarities from the PAM50 subtypes (A), methylation-based clusters (B), and multiple independent omic clustering assignments (C). (D–F) Comparison of Adjusted Rand Index for alignment between the top loadings in sample latent variables and PAM50 between other NMF algorithms and GRACKLE using regularizing similarities from the PAM50 subtypes (D), methylation-based clusters (E), and multiple independent omic clustering assignments (F) over 50 iterations.

As with the simulation studies, GRACKLE performed better with increased regularization with respect to $S_{S}$ (Fig. 4A–C). When regularizing by the PAM50 subtypes, increased gene similarity regularization with respect to $S_{G}$ also improved performance, with the top performing parameters, $\lambda_{1}$ = 50 and $\lambda_{2}$ = 16 (Fig. 4A). In contrast, regularization based on methylation clusters or other independent omic clusters showed better performance with less regularization of $S_{G}$ (highest ARI scores were observed for $\lambda_{1}$ = 90, $\lambda_{2}$ = 2 and $\lambda_{1}$ = 60, $\lambda_{2}$ = 0, respectively) (Fig. 4B and C). The top GRACKLE parameter combination routinely outperformed the other benchmarked algorithms (Fig. 4D–F). Regularization based on the PAM50 labels performed equal to regularization with methylation-based clusters (mean ARIs of 0.372 and 0.371, respectively) and much better in comparison to regularization based on multiple omic clustering labels (mean ARI of 0.261). The negative control permuted label similarities performed far below any of the benchmarked methods, indicating that sample-based regularization did indeed affect performance.

To evaluate the effects of regularization by a different GRN, we applied GRACKLE and the benchmarking algorithms using the same process as described for the TCGA analysis with PAM50 regularization using the STRING protein–protein interaction network for $S_{G}$. We found that on average the top performing parameter combination was $\lambda_{1}$ = 40, $\lambda_{2}$ = 2, indicating that increased regularization from $S_{S}$ led to better performance (Supplementary Fig. S1A). Over 50 iterations, the regularization with STRING slightly outperformed the result of regularization with the PANDA breast tissue network (Supplementary Fig. S1B).

Furthermore, to validate the observation in the TCGA data, we applied GRACKLE and the benchmarking algorithms to gene expression profiles from 1980 tumors in the METABRIC consortium. As with the TCGA analysis, we used the PAM50 labels assigned to the tumors to calculate $S_{S}$ and the PANDA breast tissue network for $S_{G}$. We assessed the performance of a 70/30 training/testing split over 50 iterations. Like the TCGA cohort, we found that the greatest alignment with PAM50 subtypes on average occurred with regularization from both the sample and gene similarities ($\lambda_{1}$ = 30, $\lambda_{2}$ = 20) (Supplementary Fig. S2A). Moreover, we found that the optimized GRACKLE results outperformed GNMF and pr-NMF (Supplementary Fig. S2B).

Overall, these results confirmed the findings from the simulation study that the sample graph regularization improves the alignment of the decomposed gene expression matrix with consideration to sample labels. Furthermore, while regularization based on sample labels from non-gene expression omic profiles performed worse than regularization with PAM50 or methylation labels, the improved performance over the other NMF algorithms demonstrates the impact of constraining NMF models using biological knowledge.

### 3.3 GRACKLE separates individuals with Down syndrome into condition-specific groups consistent with known biology

To evaluate the ability of GRACKLE to link molecule patterns to groups of diseases, we evaluated our model on the task of stratifying individuals with Down syndrome (DS) across co-occurring conditions based on their gene expression profiles. Caused by the triplication of chromosome 21 (T21), DS is the most common human aneuploidy (Mai *et al.* 2019). People with DS are at an increased risk, compared to those with disomic karyotypes, to have multiple co-occurring conditions, including hypothyroidism, sleep apnea, and autoimmune disorders, among others (Ivarsson *et al.* 1997, Sureshbabu *et al.* 2011, Dragonieri *et al.* 2022). Elucidating the underlying molecular mechanisms driving disease co-occurrence within DS is complicated by the simultaneous upregulation of the genes on chromosome 21. We evaluated if the dual regularization across sample metadata and gene networks implemented in GRACKLE could stratify samples into disease-associated groups.

Algorithm performances were evaluated by assigning samples to groups based on the top loading LV per sample and assessed for enrichment of co-occurring conditions with a one-sided fisher exact test. As with the breast cancer subtyping evaluation, we explored algorithm performance over $\lambda_{1}$ and $\lambda_{2}$ parameters ([0, 100] at an interval of 10 and [0, 20] at an interval of 2, respectively) and calculated the average performance over 50 iterations of subsampled data (70% of the cohort). We considered ranks of the reduced dimensional space, *k =* 2, *k =* 3, and *k =* 4.

**Figure 5.**
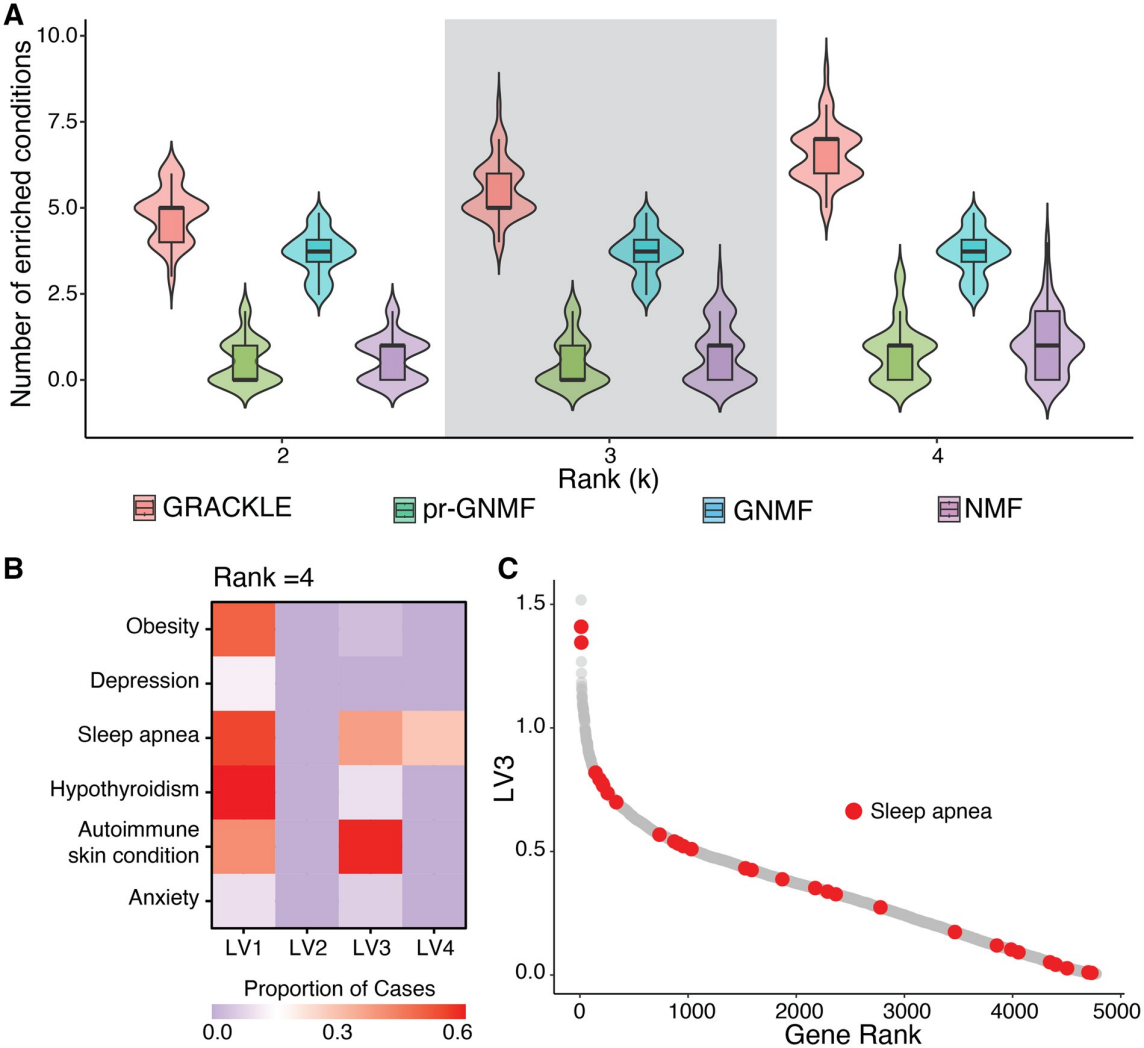
GRACKLE represents individuals with Down syndrome into condition-specific groups consistent with known biology. (A) Comparison of enriched conditions for the top loadings in sample latent variables between GRACKLE and other NMF algorithms at different ranks (*k*) over 50 iterations. (B) The proportion of enriched conditions by latent variable (LV) for the best performing parameter combination ($\lambda_{1}$ = 70 and $\lambda_{2}$ = 6) at a rank of 4. (C) Ordered gene loading weights for LV3. The highlighted nodes are genes annotated for associations with sleep apnea in the Human Phenotype Ontology.

Over the parameter sweep, GRACKLE outperformed NMF, GNMF, and pr-GNMF in identifying condition-enriched latent representations (Fig. 5A). Consistent with the previous evaluations, the top performing parameter combinations involved increased regularization by $\;S_{S}$ and relatively less regularization by $S_{G}$. As the number of ranks increased, GRACKLE identified an increased number of latent variables with enriched cases of conditions. However, as the rank increased, more individuals also had the highest loading scores for a single latent variable. Therefore, we further investigated the enrichment at a rank of *k =* 4 at a $\lambda_{1\;}$ = 70 and $\lambda_{2}$ = 6.

We found that latent variable LV1, which was the largest cluster in the four cluster solution (197 individuals) had comparatively higher proportions of individuals with a history of hypothyroidism, autoimmune skin conditions, sleep apnea, and obesity (Fig. 5B). The gene with the highest loading in LV1 was *ACIN1* (apoptotic chromatin condensation inducer 1), which is highly expressed in thyroid tissue and has been associated with immune blood cell counts (Legault *et al.* 2022). In addition, several genes in the IL6-JAK-STAT3 signaling pathway had higher loadings in LV1 (Liberzon *et al.* 2015). For example, *GRB2*, which codes for growth factor receptor-bound protein 2 had the 12th highest loading. *GRB2* was previously associated with impaired cognitive function in a mouse model of DS (Shin *et al.* 2007). As another example, the 28th ranked gene was *TNFRSF1B* (Tumor Necrosis Factor Receptor Superfamily Member 1B), which has been shown to be overexpressed in people with DS and identified as a putative causal mediator of hypothyroidism (Sullivan *et al.* 2016, 2017, Li and Gong 2023).

LV3 (32 individuals) had higher proportions of individuals with a history of autoimmune skin conditions and sleep apnea. To align the sample latent representation with the gene representation, we investigated where previously identified disease gene sets as annotated by the Human Phenotype Ontology (HPO) in the Human Molecular Signatures Database ranked across the corresponding gene loadings (Liberzon *et al.* 2015, Gargano *et al.* 2024). We found that overall, genes associated with sleep apnea ranked highly on latent variable LV3 (Fig. 5C). Though the gene with the highest loading on LV3, *SRSF4*, has not been previously associated with sleep apnea, the second and third highest loading genes, *PIGT* and *PRPS1*, were included in the HPO sleep apnea gene set.

Altogether, GRACKLE was more effective than other benchmarked algorithms in identifying subgroups of individuals that were enriched for specific co-occurring conditions. Furthermore, the aligned gene loadings were higher in known disease-associated genes.

## 4 Discussion

GRACKLE is a new framework for regularizing NMF by sample and gene similarities derived from prior information. This approach is inspired by the observation that diseases and chronic conditions are driven by changes in gene regulation. Since bulk gene expression data is high-dimensional and noisy, regularization by known gene interactions and sample annotations encourages reduced dimension representations of condition-specific mechanisms. GRACKLE has many applications, including the identification of context-specific molecular mechanisms for precise diagnostic and treatment approaches, even in small and heterogeneous cohorts.

In simulation studies, GRACKLE outperformed other NMF algorithms at increased levels of background noise present in the simulated gene expression profiles (Fig. 3). Interestingly, regularization using sample similarity, $S_{S}$, had a more pronounced effect than with gene similarity, $S_{G}$, demonstrating how impactful sample information can be when stratifying a cohort into subgroups. We tested the ability of GRACKLE to stratify breast tumor samples when regularized by gene expression, methylation, and the combination of labels from several matched omic profiles. We observed that the GRACKLE-derived latent representations were better aligned to the established PAM50 subtypes than other NMF approaches (Fig. 4). The improved alignment with matched cluster labels from other omic profiles compared to the benchmark algorithms reveals the utility of incorporating molecular measurements into the matrix decomposition. Finally, we applied GRACKLE to the stratification of individuals with T21 and found more condition-enriched subgroups than other NMF algorithms (Fig. 5).

Notably, prior biological understanding confirms the pattern of loadings for LV1 and LV3 from when GRACKLE was applied to the cohort of people with DS. People in LV1 had relatively higher proportions of individuals with a history of hypothyroidism, autoimmune skin conditions, sleep apnea, and obesity. The higher loadings for IL6-JAK-STAT3 genes correspond with the enrichment of inflammatory phenotypes (Johnson *et al.* 2018). In addition, a relatively high proportion of individuals that had the highest loadings for LV3 in the sample matrix had a history of autoimmune skin conditions and/or sleep apnea. The gene with the highest loading in this matrix was *SRSF4* which codes for the serine and arginine rich splicing factor (SRSF) 4. SRFSs are important in the regulation of alternative splicing (Graveley 2000). SRSF4 was previously identified as a biomarker for DS due to its presence in amniotic fluid supernatants only in DS fetuses (Tsangaris *et al*. 2006). Furthermore, alterations in SRSF4 function have been associated with systemic autoimmunity and neurodegenerative disorders, including Alzheimer’s disease in people with DS (Li *et al.* 2023).

There remain several areas to improve GRACKLE. First, in the simulation studies, we only simulated modules of stochastic upregulation to represent stimulation of pathway activity. Simulations with both up and downregulation will be the subject of future studies. Second, to identify the optimum set of regularization parameters, we performed a grid search over a range of values for $\lambda_{1}$ and $\lambda_{2}$ across multiple subsets of data. If the ground truth (i.e. sample group labels) is unknown, we suggest that a user performs *k*-means clustering over the sample metadata and performs a grid search over $\lambda_{1}$ and $\lambda_{2}$ parameters ([0, 100] at an interval of 10 and [0, 20] at an interval of 2, respectively). However, future directions will test other measures to guide unbiased parameter selection.

Furthermore, we chose to benchmark GRACKLE against three NMF algorithms: NMF, which learns a representation solely on the data, GNMF, which applies regularization based on a learned sample similarity matrix of the data using *k*-nearest neighbors, and pr-GNMF, which regularizes based on prior knowledge about gene–gene interactions (much like netNMF-sc does with known gene–gene co-expression). We chose benchmark methods that provide latent representations in both the sample and gene space. Deep learning models could provide an interesting comparison in both performance and interpretability of results. Additionally, methods such as nNMF (Chalise *et al*. 2020), DMCL (Chen *et al*. 2023), and MLMF (Ren *et al*. 2024) regularize with respect to sample similarity, but do not include the simultaneous regularization using a GRN. Future benchmarking will evaluate the benefits of the regularization with respect to GRNs compared to these methods.

The sample similarity, $S_{S}$, and gene similarity, $S_{G}$, matrices are critical inputs to GRACKLE and there are many ways to calculate either matrix. While we have tested and benchmarked several ways to calculate similarity between samples, there is potential in calculating similarity to reflect patient outcomes, such as response to treatments, disease progression, or other metrics of survival. The GRACKLE framework could also handle multiple measurements from different time points. Moreover, combining multiple forms of metadata, as was done with multiple clustering assignments across several omics in the breast tumor stratification application, creates a sample similarity that is less sensitive to any single assignment and can handle missing data. An open question is how robust is GRACKLE to missing values across the input gene expression or sample metadata?

Considering gene similarity, we noted that regularization with respect to $S_{G}$ was less impactful in GRACKLE than regularization by $S_{S}$. Several differences exist between $S_{S}$ and $S_{G}$ gene similarity. For example, considering the TCGA PAM50 subtypes, there is a great discrepancy in the transitivity of the graphs ($S_{S}$ = 1, $S_{G}$ = 0.002). As another measure, the graph entropy of $S_{S}$ is 1.84, while the entropy of $S_{G}$ is 4.82. This indicates that there is higher uncertainty and lower organization in $S_{G}$ compared to $S_{S}$.

We found improved performance with increased regularization by $S_{G}$ in the breast tumor stratification where $S_{S}$ constructed with PAM50 labels in the TCGA and METABRIC applications. We hypothesize that in this positive control, even a little regularization by $S_{S}$ aligned with the ground truth and thus was less affected by $S_{G}$. Further benchmarking is required to explain this phenomenon.

As we saw with the gene similarities derived from PANDA or STRING networks, GRACKLE is flexible to how $S_{G}$ is calculated, and results were strong using both network models. A robust evaluation of different gene–gene similarities will be the subject of future studies. We anticipate that there are opportunities for both gains in performance and interpretation of the model output with different $S_{G}$ matrices as was shown in the analysis using the STRING network. Additionally, GRACKLE can be applied to metabolomics, proteomics, or any other data modalities.

Identifying pathways and processes that underlie a group of samples or patients is a critical challenge in biological and biomedical research. GRACKLE is a novel solution to this challenge that offers both flexibility and interpretability. There are numerous application areas for GRACKLE, along with many opportunities for the method to be further developed.

## Supplementary Material

btaf213_Supplementary_Data

## Data Availability

Data and code for the simulation studies are available at https://github.com/lagillenwater/GRACKLE. TCGA breast tumor RNASeq expression profiles and metadata were downloaded using the TCGAbiolinks R package (version 2.34.0). These data are also available at https://portal.gdc.cancer.gov/. Clinical profiles from the Human Trisome project cohort are available on both the Synapse data sharing platform (https://doi.org/10.7303/syn31488784) and the INCLUDE Data Hub (https://portal.includedcc.org/). Whole blood transcriptome data for the participants with Down syndrome are accessible through Synapse (https://doi.org/10.7303/syn31488780), the INCLUDE Data Hub, and Gene Expression Omnibus (GSE190125).
