# Supplementary material for: GRACKLE: an interpretable matrix factorization approach for biomedical representation learning
Source: Bioinformatics. 2025 Jul 15;41(Suppl 1):i609–18. doi: 10.1093/bioinformatics/btaf213 (PMC12261436; doi:10.1093/bioinformatics/btaf213)
Supplement: btaf213_Supplementary_Data [file btaf213_supplementary_data.zip › btaf213_Supplementa/Gillenwater.384.sup.pdf]

Supplemental Material for

GRACKLE: An interpretable matrix factorization approach for biomedical representation learning.

Lucas A Gillenwater[1,2,3*], Lawrence E Hunter[4], James C Costello[1,2,3,5*]
[1]Department of Pharmacology, University of Colorado Anschutz Medical Campus, Aurora, CO
[2]Computational Bioscience Program, University of Colorado Anschutz Medical Campus, Aurora, CO
[3]Linda Crnic Institute for Down syndrome, University of Colorado Anschutz Medical Campus, Aurora, CO
[4]Department of Pediatrics, University of Chicago, Chicago, IL
[5]Department of Biomedical Informatics, University of Colorado Anschutz Medical Campus, Aurora, CO
*Corresponding authors. E-mail: lucas.gillenwater@cuanschutz.edu; james.costello@cuanschutz.edu

Supplemental Figures

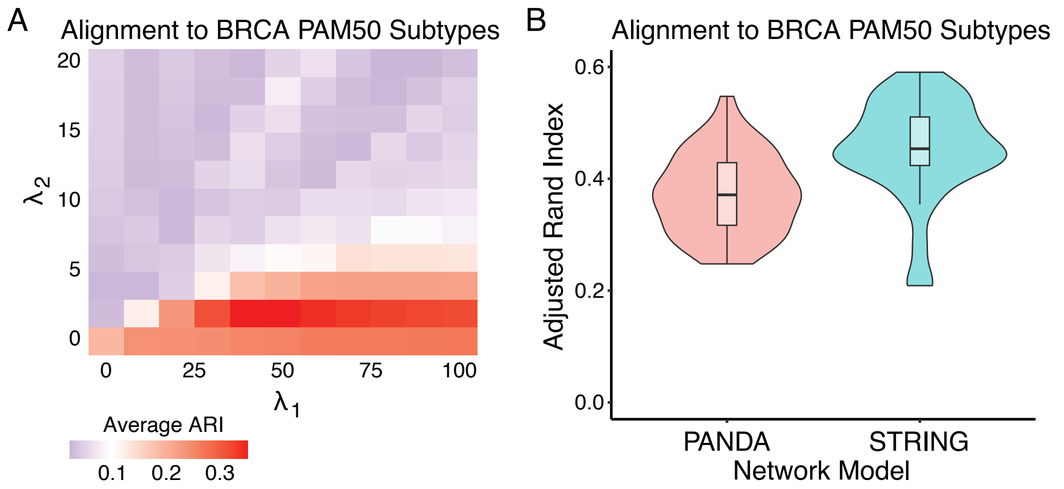

Figure S1. The effect of different network model similarities. (**A**) Grid search of the average adjusted rand index (ARI) over 50 iterations for alignment between the top loadings in sample latent variables and PAM50 subtypes from the TCGA breast cancer dataset (Koboldt *et al.* 2012) over λ_1 and λ_2 parameters regularized using the PAM50 subtypes (sample similarity) and the STRING (Szklarczyk *et al.* 2023) network model to calculate gene similarities. (**B**) Comparison of Adjusted Rand Index of latent variables assigned to TCGA tumor gene profiles based on top loadings and PAM50 subtypes between the optimized GRACKLE results when regularized with sample similarities calculated from the PAM50 subtypes and gene similarities derived from either the PANDA (Glass *et al.* 2013) or STRING network models.

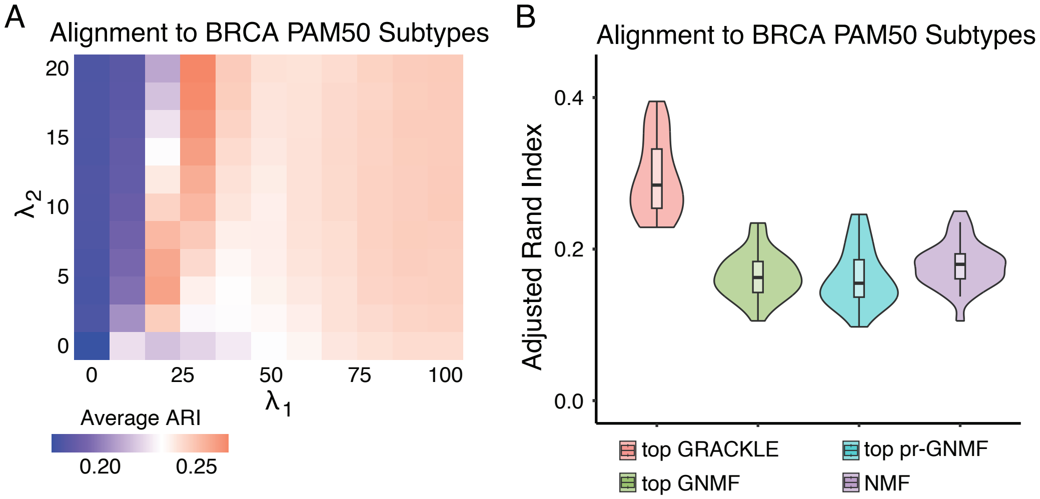

Figure S2. Model performance on predicting PAM50 subtype labels using the METABRIC breast cancer dataset. (Curtis *et al.* 2012) (**A**) Grid search of the average adjusted rand index (ARI) over 50 iterations for alignment between the top loadings in sample latent variables and PAM50 subtypes over λ_1 and λ_2 parameters regularized using the PAM50 subtypes (sample similarity) and the PANDA network model to calculate gene similarities. (**B**) Comparison of Adjusted Rand Index of latent variables assigned to METABRIC tumor gene profiles based on top loadings and PAM50 subtypes between the optimized GRACKLE results when regularized using sample similarities calculated from the PAM50 subtypes from METABRIC and gene similarities derived from the PANDA network model.

References

Curtis C, Shah SP, Chin S-F *et al.* The genomic and transcriptomic architecture of 2,000 breast tumours reveals novel subgroups. *Nature* 2012;**486**:346–52.

Glass K, Huttenhower C, Quackenbush J *et al.* Passing messages between biological networks to refine predicted interactions. *PLoS One* 2013;**8**:e64832.

Koboldt DC, Fulton RS, McLellan MD *et al.* Comprehensive molecular portraits of human breast tumours. *Nature* 2012;**490**:61–70.

Szklarczyk D, Kirsch R, Koutrouli M *et al.* The STRING database in 2023: protein–protein association networks and functional enrichment analyses for any sequenced genome of interest. *Nucleic Acids Research* 2023;**51**:D638–46.
